# Hematopoietic Stem Cell Cytokines and Fibroblast Growth factor-2 Stimulate Human Endothelial Cell-Pericyte Tube Co-Assembly in 3D Fibrin Matrices under Serum-Free Defined Conditions

**DOI:** 10.1371/journal.pone.0085147

**Published:** 2013-12-31

**Authors:** Annie O. Smith, Stephanie L. K. Bowers, Amber N. Stratman, George E. Davis

**Affiliations:** Department of Medical Pharmacology and Physiology, Dalton Cardiovascular Sciences Center and University of Missouri School of Medicine, Columbia, Missouri, United States of America; University of Illinois at Chicago, United States of America

## Abstract

We describe a novel 3D fibrin matrix model using recombinant hematopoietic stem cell cytokines under serum-free defined conditions which promotes the assembly of human endothelial cell (EC) tubes with co-associated pericytes. Individual ECs and pericytes are randomly mixed together and EC tubes form that is accompanied by pericyte recruitment to the EC tube abluminal surface over a 3-5 day period. These morphogenic processes are stimulated by a combination of the hematopoietic stem cell cytokines, stem cell factor, interleukin-3, stromal derived factor-1α, and Flt-3 ligand which are added in conjunction with fibroblast growth factor (FGF)-2 into the fibrin matrix. In contrast, this tube morphogenic response does not occur under serum-free defined conditions when VEGF and FGF-2 are added together in the fibrin matrices. We recently demonstrated that VEGF and FGF-2 are able to prime EC tube morphogenic responses (i.e. added overnight prior to the morphogenic assay) to hematopoietic stem cell cytokines in collagen matrices and, interestingly, they also prime EC tube morphogenesis in 3D fibrin matrices. EC-pericyte interactions in 3D fibrin matrices leads to marked vascular basement membrane assembly as demonstrated using immunofluorescence and transmission electron microscopy. Furthermore, we show that hematopoietic stem cell cytokines and pericytes stimulate EC sprouting in fibrin matrices in a manner dependent on the α5β1 integrin. This novel co-culture system, under serum-free defined conditions, allows for a molecular analysis of EC tube assembly, pericyte recruitment and maturation events in a critical ECM environment (i.e. fibrin matrices) that regulates angiogenic events in postnatal life.

## Introduction

 There continues to be a great need for studies concerning the fundamental cell biology of how blood vessels form, mature and stabilize [1–12]. Many critical issues regarding our understanding of these events have been solved using a variety of approaches and most notably by *in vitro* systems of vascular morphogenesis and maturation in 3D matrix environments. For example, major advances have occurred in our understanding of how ECs form tubes during morphogenic events and how pericytes recruit to tubes and regulate tube remodeling as well as stimulate maturation events such as vascular basement membrane matrix assembly [1,4,7–9,13–15]. Furthermore, recent work has illustrated that complex vascular morphogenic and maturation processes can be accomplished with isolated cells in 3D matrix systems under serum-free defined conditions, an approach that our laboratory has performed for many years [13,14,16–19]. By far the majority of *in vitro* studies with endothelial cells utilize serum-containing media. Under these conditions, it is very difficult (if not impossible) to define the growth factor, peptide, hormone, and lipid requirements for a given biological event that is being examined. 

 A number of high quality endothelial cell morphogenic systems in 3D matrices have been developed over the years using either isolated human endothelial cells [17–22] or using biologic tissues such as pieces of vessels including rodent aorta [23,24] . A true test of the quality and merit of any given system is what can be accomplished with these systems over time and it is pretty evident which systems have been utilized that have significantly advanced our understanding of vascular morphogenesis including lumen formation and sprouting [15,16,18–21,23,25–36] as well as the functional ability of pericytes to modulate tube formation and maturation (and including the dynamic nature of these events by performing and analyzing real-time movies) [4,9,13,15,37–39]. Some of the same models have also advanced our understanding of important processes such as vascular tube regression as well as the ability of pericytes to prevent pro-regressive stimuli, by presenting molecules such as TIMP-3 [13,15,40–43]. An additional point is that the systems that have worked particularly well have been performed in either 3D collagen or fibrin matrices, which are the two major extracellular matrix environments where vascular morphogenesis takes place [8,44].

 In this work, we report an important advance in the ability to perform 3D fibrin vascular morphogenic assays with isolated human ECs and pericytes under serum-free defined conditions. We demonstrate that the hematopoietic cytokines, stem cell factor (SCF), interleukin-3 (IL-3), stromal-derived factor (SDF)-1α in conjunction with fibroblast growth factor (FGF)-2 stimulate EC-pericyte tube co-assembly in 3D fibrin matrices. The addition of Flt-3 ligand (Flt-3L) further stimulates this process. We performed these assays in a microwell format, performed real-time movies of these events and demonstrated both tubulogenesis and sprouting in response to the combination of hematopoietic stem cell cytokines and FGF-2. Furthermore, we showed that pericyte recruitment to EC tubes leads to vascular basement membrane matrix deposition and EC-pericyte tube co-assembly as well as sprouting that were dependent on the α5β1 integrin. Thus, this novel system will be particularly useful to elucidate fundamental mechanisms underlying EC tubulogenesis, sprouting, and pericyte-induced maturation events in 3D fibrin matrices, a critical matrix environment regulating postnatal angiogenesis.

## Materials and Methods

### Reagents

The fibrin matrix consisted of human plasminogen-depleted fibrinogen (EMD Chemicals, Billerica, MA), and human plasma fibronectin (FN) (Sigma-Aldrich, St. Louis, MO). For select basement membrane experiments, bovine fibronectin (Sigma-Aldrich) was utilized. The following cytokines and growth factors were added to the gels: recombinant human stromal-derived factor-1α, stem cell factor, interleukin-3, Flt-3 ligand and fibroblast growth factor (FGF-2) (R&D Systems, Minneapolis, MN). Fibrinogen gels were catalyzed by thrombin addition (Sigma-Aldrich) in 96 well full-area assay plates (Costar, Corning, NY). For each experiment, the defined media consisted of: 1xM199 (Gibco, Grand Island, NY), FGF-2, reduced serum supplement II (RSII) [18], ascorbic acid (AA) (Sigma-Aldrich) and aprotinin (Sigma-Aldrich). For integrin blocking experiments, α_1_-α_5,_ αV, αVβ3, αVβ5 and α1β1 integrin-blocking antibodies were from EMD Millipore (Billerica, MA) ; α_6_ blocking antibody (Abcam, Cambridge, MA). Blocking mouse monoclonal antibodies directed to IL-3, SDF-1α, and VEGF as well as blocking goat polyclonal antibodies directed to SCF, PDGF-BB and HB-EGF were from R&D Systems. Imatinib was from Cayman Chemical (Ann Arbor, MI) and gefitinib was from Tocris (Bristol, UK). Primary immunostaining antibodies were as follows: CD31 (Dako, Carpinteria, CA) laminin (Sigma-Aldrich), fibronectin (Sigma-Aldrich), collagen type I (Sigma-Aldrich), collagen type III (Sigma-Aldrich), collagen type IV (EMD Millipore), fibrinogen (Sigma-Aldrich), nidogen 1 and 2 (R&D Systems), perlecan (Novex, Grand Island, NY). Secondary immunostaining antibodies were as follows: Goat anti-Rabbit IgG, Rabbit anti-Goat IgG, Rabbit anti-Mouse IgG (Molecular Probes, Grand Island, NY).

### Cell Culture

Human umbilical vein ECs (HUVECs) were purchased from Lonza (Walkersville, MD) and passages 3 to 6 were used for all experiments. AcGFP-F ECs and mCherry EC cell lines were recently developed and described [36] and used for videomicroscopy experiments from passages 3 to 6. Human brain vascular pericytes were used from passages 4-10 [14]. Both GFP and mCherry labeled pericytes (PC) were used. ECs were cultured in 1xM199, 20% FBS (Sigma-Aldrich), 400 µg/ml endothelial cell growth supplement (ECGM) extracted from bovine hypothalamus (Pel-freeze, Rogers, AR), 100 µg/ml heparin sodium salt (Sigma-Aldrich), 1% antibiotic-antimycotic (Gibco), and 10 µg/ml gentamicin (Gibco). PCs were grown in 1X DMEM (Gibco) with 10% FBS. For many experiments, ECs were primed overnight (16-20 hr) using RSII-containing media with 40 ng/ml of FGF-2 and human VEGF 165 (R&D Systems) in 9mls 1xM199.

### 3D Fibrin Vasculogenic Assays

Fibrin gels were prepared using plasminogen-depleted human fibrinogen in 1x PBS at a concentration of 5 mg/ml. The fibrinogen was warmed to room temperature and filtered for sterility. Human plasma fibronectin was added to a concentration of 100 µg/ml. The following hematopoietic cytokines and growth factors were added at a concentration of 200 ng/ml: SCF, IL-3, Flt-3L, SDF-1α, and FGF-2. Endothelial cells were added at 2x10^6^ cells/ml while pericytes were added at 0.4x10^6^ cells/ml within the fibrin matrices and thoroughly mixed. Thrombin was used to catalyze the fibrinogen gel at a final concentration of 0.25 µg/ml. The fibrinogen-cell-growth factor mixture was aliquoted in 50 µl on top of 1 µl dots of thrombin in 96 well full area plates. Prior to fibrin polymerization, the plates were gently tapped on all four edges to assure that the fibrin gel is equally distributed throughout the well. All empty wells were filled with sterile water in order to hydrate the plates. After 30 minutes incubation at 37°C, gels were fed with 200 µl defined media including RSII, FGF-2 at 40 ng/ml, AA at 50 µg/ml, and aprotinin (used at 1:4000 dilution) all in 1xM199. An 18 gauge sterile needle was used to carefully clear any bubbles from between the gel and media. After 3 or 5 days of culture, assay plates were fixed with 2% paraformaldehyde for at least one hour before immunostaining or quantitative analysis was performed. For invasion assays, pericytes were added into the fibrin matrix at 1x10^6^ cells/ml with all of the factors as described above. After incubation for 30 min. at 37°C, the ECs were mixed with the defined media and seeded on top of the gels at a concentration of 100,000 cells/well. For 3 days cultures, 120 µl of media was removed on day 2, which was then replaced by 120 µl of fresh media prepared as above. For 5 days cultures, the replacement of media occurred on day 2 and day 4. Real-time movies were performed as described using a Leica inverted fluorescence microscope equipped with an environmental chamber to control temperature, CO_2_ and humidity [18]. 

### Immunostaining of Cultures

Fixed fibrin gels were gently removed with clean fine forceps into 48 well plates with 500 µl Tris-glycine solution. These were rocked at 4°C for 2 hours. Next, the Tris-glycine was removed and 500 µl antibody specific blocking solutions that were Tris-buffered saline (TBS) containing 1% bovine serum albumin (with added 1% serum of the species of the secondary antibody) for an additional 2 hours. Primary antibodies were then added directly to the wells with blocking solution and incubated overnight at 4°C. For 2-3 hours, gels were sufficiently washed with TBS and 500 µl blocking solution was again added with the addition of secondary antibodies for at least 3 hours. Final washes with TBS were performed over several hours to days, after which the cultures were examined by immunofluorescence microscopy.

### Electron Microscopy

A 3% glutaraldehyde fixative solution was prepared with 1 ml electron microscopy grade glutaraldehyde (Sigma-Aldrich), 4.17 ml 2xM199, 3.16 ml sterile ddH_2_O, and ~7 µl 5N NaOH. Cultures were fixed with 200 µl 3% glutaraldehyde solution. Plates were incubated at 37°C for 1 hour, washed generously with 1xM199 and stored in 1xM199 at 4°C. Samples were processed at the Electron Microscopy Core Facility at the University of Missouri-Columbia, and images were acquired using a JEOL 1400 transmission electron microscope (TEM). 

### Microscopy and Imaging

Time-lapse videomicroscopy was performed using a fluorescence inverted microscope (DMI6000B, Leica). Fluorescence imaging was also performed using a fluorescence inverted microscope (Eclipse TE2000-E; Nikon), and the analysis software MetaMorph (Molecular Devices) was used to quantitate EC tube area during the morphogenic process as described [18]. 

### Statistical Analysis

Statistical analysis of selected EC vasculogenic and lumen formation data was performed using Microsoft Excel (Microsoft). Statistical significance was set at minimum with *P* less than 0.05. Student *t* tests were used when analyzing 2 groups within a single experiment (with n ≥ 10). 

## Results

### Development of a novel human endothelial cell-pericyte co-culture system under serum-free defined conditions in 3D fibrin matrices

**Figure 1:**
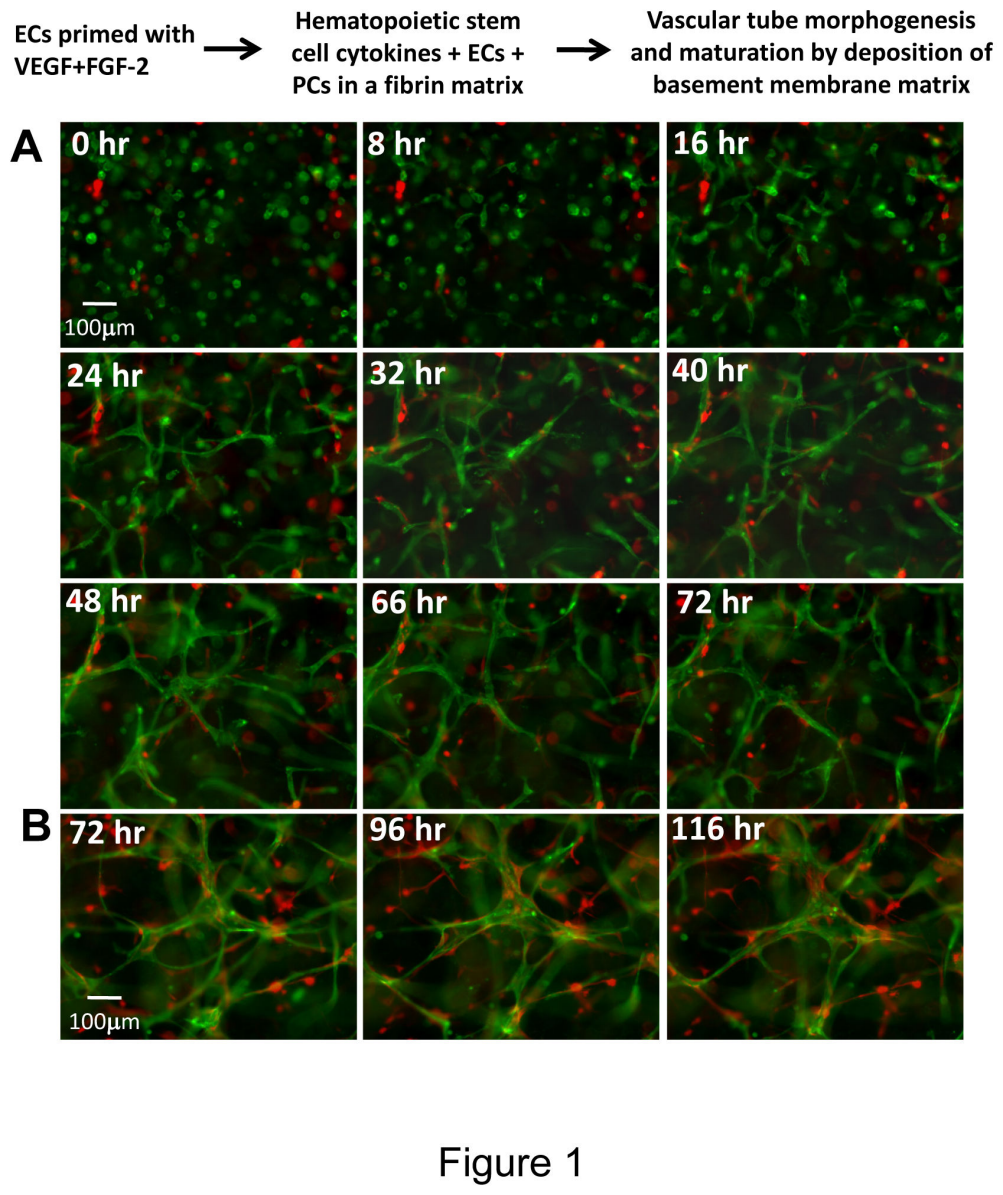
Real-time images showing endothelial cell tube formation and pericyte recruitment using a serum-free defined system in 3D fibrin matrices. Hematopoietic stem cell cytokines and FGF-2 stimulate these morphogenic responses which can be primed by EC pre-treatment with VEGF and FGF-2. Endothelial cells (ECs) were labeled with membrane targeted GFP while pericytes (PCs) were labeled with mCherry. The two cell types were randomly mixed into fibrin matrices in the presence of SCF, IL-3, SDF-1α, Flt-3L and FGF-2. Prior to harvesting the cells for suspension into fibrin gels, ECs were primed with VEGF and FGF-2 for 20 hr. After fibrin polymerization, cultures were imaged every 10 minutes and select fluorescent images are shown at the indicated times. Two different series (A,B) are illustrated. Bar equals 100 µm.

**Figure 2:**
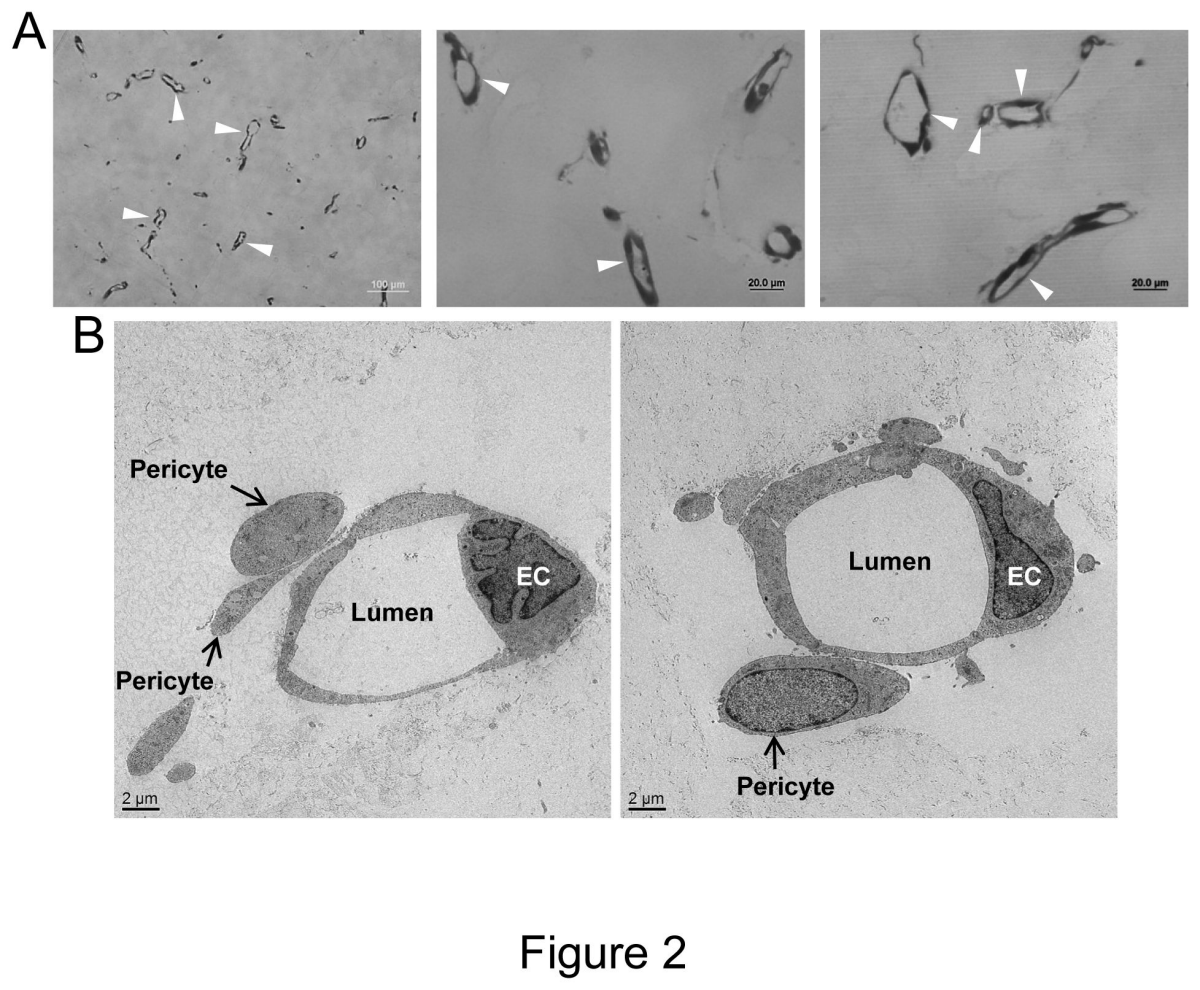
Cross-sections of EC-pericyte co-cultures reveal marked EC tubulogenesis using a serum-free defined system in 3D fibrin matrices. (A) EC-pericyte co-cultures were established and after 5 days, cultures were fixed, embedded into plastic, cross-sectioned, stained with toluidine blue, and photographed. Arrowheads indicate EC lumens in cross-section. (B) EC-pericyte co-cultures were established for 5 days, fixed and processed for transmission electron microscopy. EC lumen formation is observed and pericytes are demonstrated on the EC abluminal surface (arrows).

In this work, we sought to develop a new system in which vascular tube morphogenesis and pericyte recruitment occurred in 3D fibrin matrices under serum-free defined conditions and using 96 well microplates (which allows for many manipulations and conservation of materials and cells). We and others have previously described 3D fibrin models using endothelial cells (ECs) alone and media which contained serum. However, under these conditions, it is not clear what growth factor and/or matrix requirements are necessary for these events. Furthermore, previous systems have not utilized pericytes which support developing capillary tubes. Previous work from our laboratory in collagen matrices have characterized the functional role of pericytes (using human or bovine pericytes) during capillary tube maturation and have demonstrated their ability to recruit to EC-lined tubes and to stimulate maturation events such as vascular basement membrane assembly [9,13,14]. However, assays such as these have not been developed in 3D fibrin matrices under defined serum-free conditions. Thus, using defined recombinant growth factors in serum-free media, we have developed a human EC-pericyte tube co-assembly model in 3D fibrin matrices (Figure 1). This is illustrated using real-time video microscopy with representative still images (Figure 1) and movies that demonstrate the EC-pericyte tube co-assembly process (Videos S1-S4). In this assay, ECs are labeled with membrane-tagged AcGFP and pericytes are labeled with mCherry. ECs were primed with vascular endothelial growth factor (VEGF) and fibroblast growth factor (FGF)-2 for 20 hr prior to the culture set-up. GFP-ECs and mCherry-pericytes were randomly mixed in 3D fibrin matrices and in the presence of added stem cell factor (SCF), interleukin-3 (IL-3), stromal-derived factor-1α (SDF-1α), Flt-3 ligand (Flt-3L) and FGF-2. Cultures were established at a 4:1 EC:pericyte ratio and were imaged using real-time fluorescence microscopy over a 5 day period (Figure 1, Videos S1-S4). As indicated in the still images and real-time movies, there is dramatic EC tube formation with pericyte recruitment to EC-lined tubes and subsequent pericyte motility along the tube abluminal surface which resemble capillary tubes observed *in vivo*. To confirm that these structures are indeed tubes, we embedded fixed gels in plastic and prepared thin sections that were observed by both light microscopy and transmission electron microscopy. As shown in Figure 2A, marked EC tube formation is observed in cross-section (arrowheads) by light microscopy and electron microscopy. In addition, EC tubes are observed with associated pericytes (arrows) that have recruited to the EC abluminal surface (Figure 2B). Thus, we have established serum-free defined conditions that allow human ECs and pericytes to co-assemble into anatomically correct capillary tube structures with abluminally associated pericytes in 3D fibrin matrices. 

### Hematopoietic stem cell cytokines in conjunction with FGF-2 stimulate human vascular tube morphogenesis in 3D fibrin matrices under defined serum-free conditions

**Figure 3:**
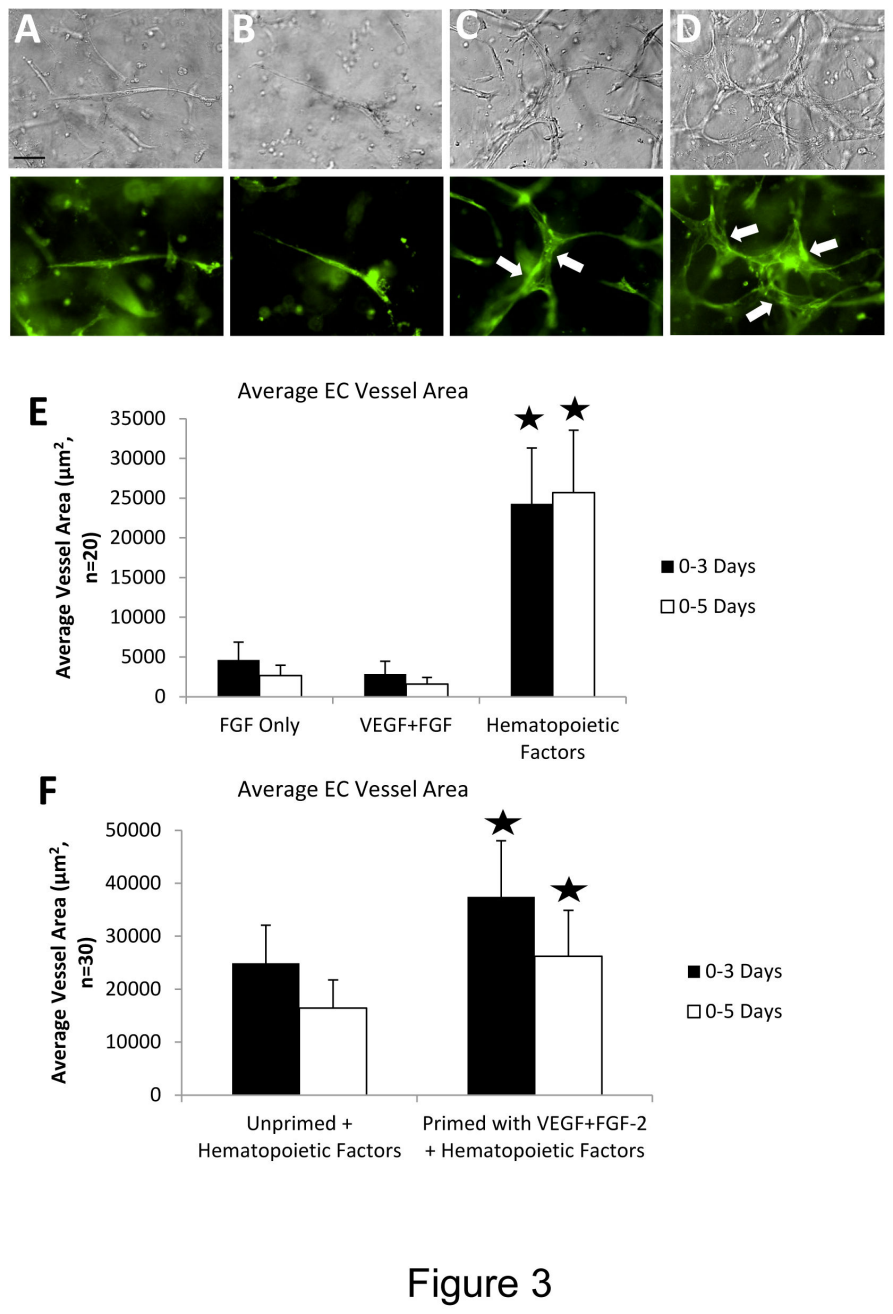
Hematopoietic stem cell cytokines stimulate EC-pericyte tube co-assembly in 3D fibrin matrices. Membrane-targeted GFP labeled ECs were mixed with pericytes in 3D fibrin matrices with various growth factor combinations. The upper panels (A-D) show light microscopic images while the lower panels show fluorescent images of the corresponding light image fields from cultures at 72 hr. (A) FGF-2 only; (B) VEGF+FGF-2; (C) Hematopoietic factors including SCF, IL-3, SDF-1α were added along with FGF-2; (D) Same factors added as C during the assay, but ECs were primed first with VEGF and FGF-2 overnight. Arrows indicate EC-lined tubes. Bar equals 25 µm. (E,F) EC-pericyte co-cultures were established with unprimed (E) or VEGF/FGF-2 primed (F) ECs in 3D fibrin matrices with the indicated growth factors. Cultures were fixed after 72 or 120 hr and tube area was quantitated using Metamorph software. Asterisks indicate significance of p< .01 relative to the FGF only condition (E) (n=20, values are averaged + SD) or relative to the unprimed+hematopoietic factors condition (F) (n=30, values are averaged + SD).

**Figure 4:**
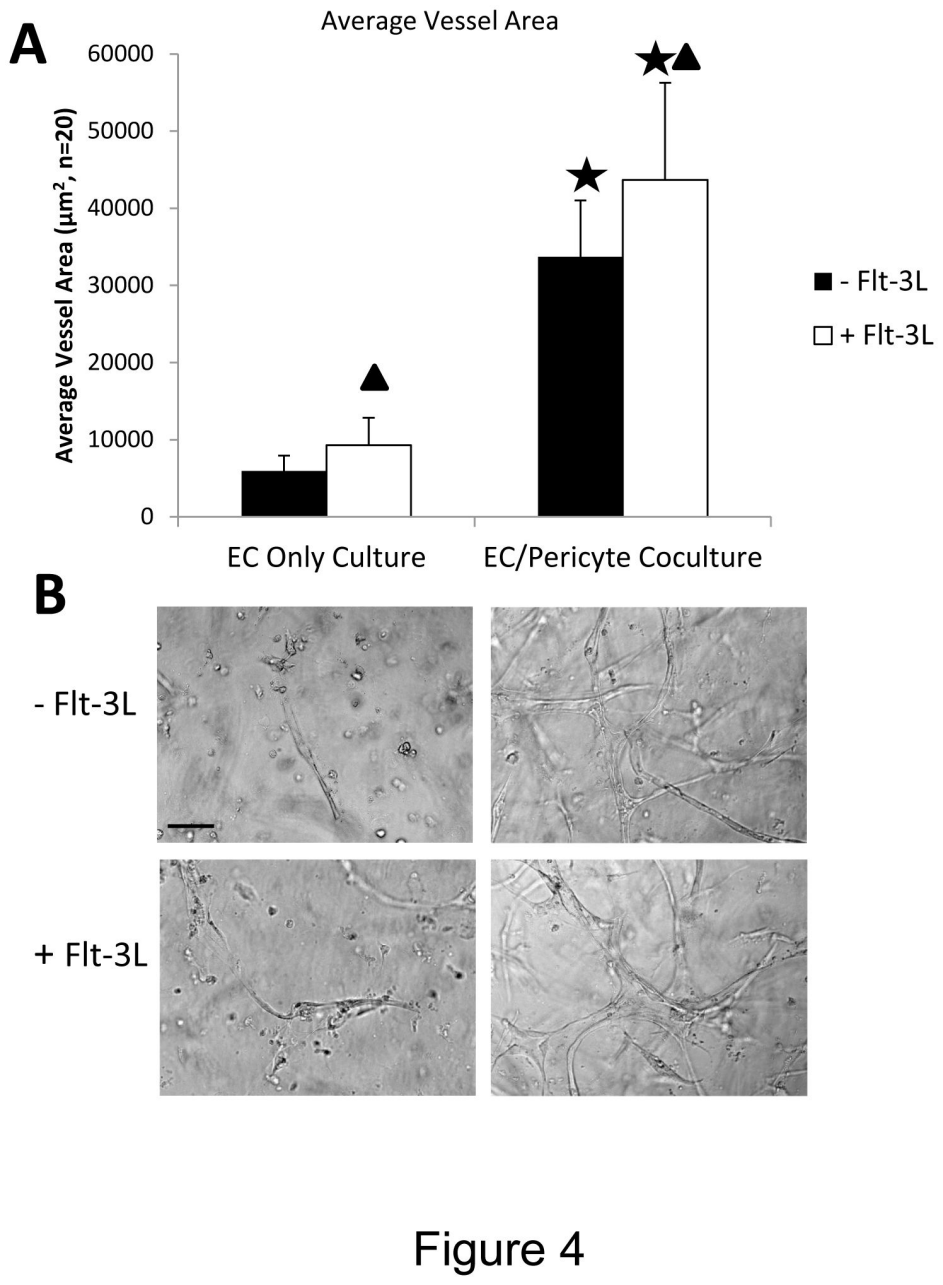
Pericytes and Flt-3L enhance the ability of SCF, IL-3, SDF-1α and FGF-2 to stimulate EC tubulogenesis in 3D fibrin matrices. ECs were seeded with or without pericytes and hematopoietic stem cell cytokines (SCF, IL-3, SDF-1α, FGF-2) were added into the fibrin matrices in the presence or absence of Flt-3L. After 72 hr, cultures were fixed, photographed (B), and quantitated for EC tube formation using Metamorph Software (A). Asterisks indicate significance at p< .01 with respect to the EC only cultures, while the triangles indicate significance at p< .01 with respect to cultures without Flt-3L addition (n=20, values are averaged + SD). Bar equals 25 µm.

To address which growth factors are required for vascular tube morphogenesis in 3D fibrin matrices under serum-free defined conditions, we tested combinations of recombinant growth factors to attempt to induce this process. A central assumption for many years has been that the combination of VEGF and FGF isoforms, such as FGF-2, are responsible for the processes of EC tube formation and sprouting in 3D matrices. Recently, we demonstrated that this combination of growth factors failed to support human EC tubulogenesis, EC sprouting or EC-pericyte tube co-assembly in 3D collagen matrices under defined serum-free conditions [16]. In contrast, the combination of SCF, IL-3, SDF-1α, and FGF-2 strongly supported EC tubulogenesis and EC-pericyte tube co-assembly in 3D collagen matrices [16]. Here, we demonstrate that the same combination of hematopoietic cytokines (i.e. SCF, IL-3, and SDF-1α) with the addition of Flt-3L and FGF-2 optimally stimulates EC tube formation and EC-pericyte tube co-assembly in 3D fibrin matrices (Figures 3 and 4). The addition of Flt-3L is not necessary for these processes to occur, but it does enhance these events along with the addition of pericytes, which likely protects the fibrin matrix from proteolytic degradation (Figure 4). Importantly, the addition of FGF-2 alone or VEGF and FGF-2 together fail to support EC tube formation or EC-pericyte tube co-assembly in 3D fibrin matrices (Figure 3). Thus, we are showing that a critical extracellular matrix facilitator of vascular tube formation (i.e. fibrin matrices), which is fundamentally important in postnatal angiogenic events, depends on combinations of hematopoietic stem cytokines to stimulate vessel formation (Figures 3 and 4). 

### VEGF and FGF-2 prime EC tube morphogenesis and EC-pericyte tube co-assembly in response to hematopoietic stem cell cytokines in 3D fibrin matrices under serum-free defined conditions

Previously, we reported that VEGF’s primary function in our defined assay system in collagen matrices was to prime or prepare ECs for subsequent tube morphogenesis which is directly stimulated by hematopoietic stem cell cytokines [16]. FGF-2 alone as well as the combination of VEGF and FGF-2 (which caused the greatest effect), were capable of priming EC tube morphogenesis in response to hematopoietic cytokines [16]. To test whether VEGF and FGF-2 could prime EC tube morphogenesis in 3D fibrin matrices, we performed this experiment (Figure 3F) which demonstrates that these growth factors can indeed prime these events. An important point is that the tube morphogenic process occurs in the absence of VEGF priming (Figure 3F), but clearly VEGF does prime morphogenesis which results when hematopoietic cytokines and FGF-2 are added into the 3D fibrin matrices.

**Figure 5:**
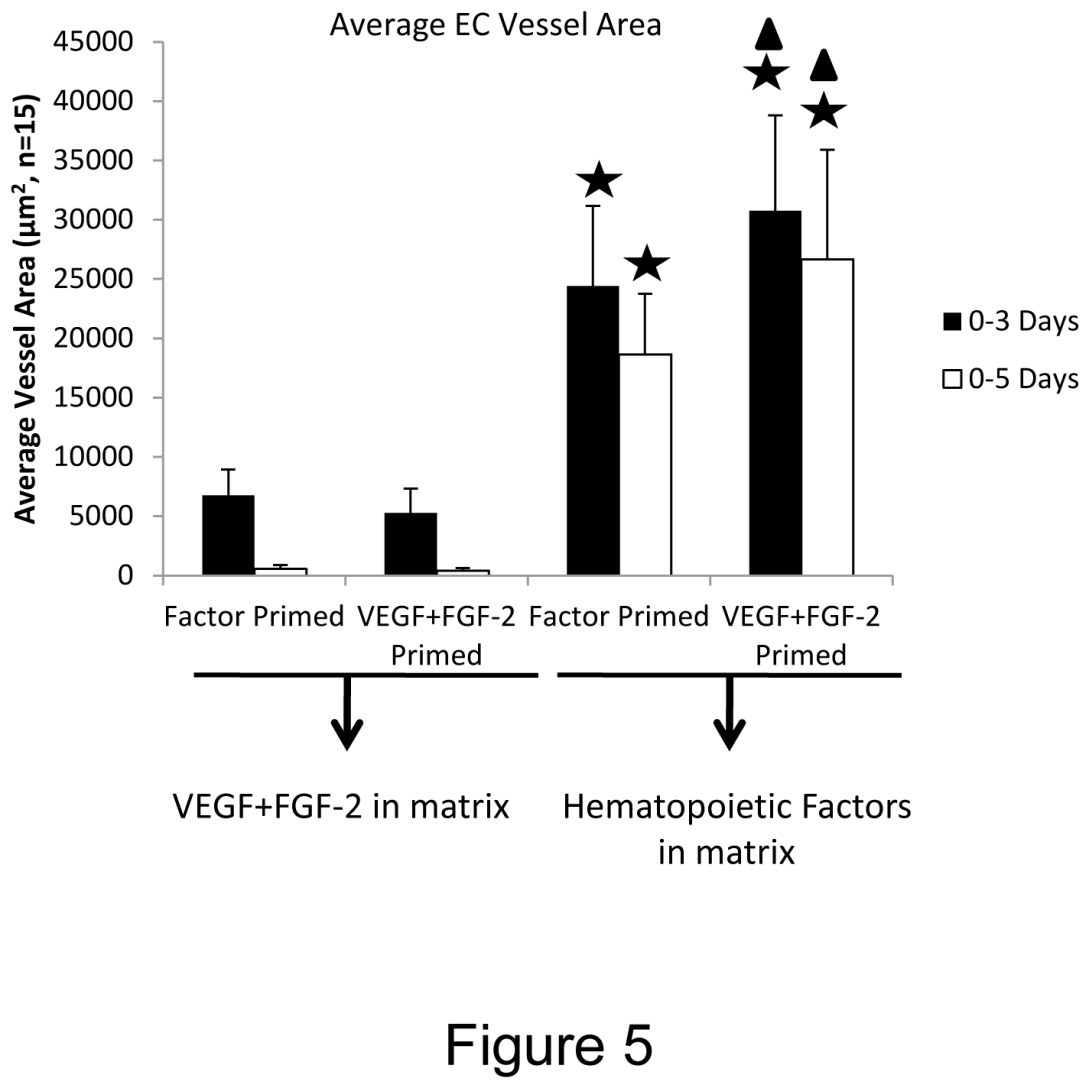
VEGF and FGF-2 prime ECs upstream of hematopoietic stem cell cytokines which act downstream to control EC tubulogenesis and EC-pericyte tube co-assembly in 3D fibrin matrices. ECs were primed with hematopoietic cytokines (factors) or VEGF/FGF-2 overnight and then were seeded into fibrin matrices with hematopoietic factors including FGF-2 or with VEGF/FGF-2 alone so that all four possible combinations were examined. Pericytes were co-seeded with the hematopoietic factor or VEGF/FGF-2 primed ECs. Cultures were fixed after 72 or 120 hr and EC tube area was measured using Metamorph software. Asterisks indicate significance at p< .01 with respect to the Factor primed/ VEGF+FGF-2 in matrix conditions, while the triangles indicate significance at p< .05 with respect to the Factor primed/Hematopoietic factors in matrix conditions (n=15, values are averaged + SD).

In addition, we addressed whether the order of addition of the priming versus morphogenic stimuli would affect the tube morphogenic process in 3D fibrin matrices (Figure 5). Previously, we reported that VEGF priming was needed upstream of hematopoietic cytokine addition in order for its effect to be observed (due in part to the upregulation of hematopoietic cytokine receptors such as IL-3 receptor alpha) [16]. VEGF/FGF-2 priming of ECs followed by supplying VEGF/FGF-2 during the morphogenic assay failed to lead to tube morphogenesis in 3D collagen matrices [16]. Here, we have primed ECs with VEGF/FGF-2 vs. hematopoietic cytokines and then added either VEGF/FGF-2 or hematopoietic cytokines/FGF-2 into the fibrin matrices in order to address this question (Figure 5). The addition of hematopoietic cytokines and FGF-2 into the fibrin matrices are clearly required for EC tube formation and EC-pericyte tube co-assembly no matter whether a priming stimulus was provided or not (Figure 5). Furthermore, VEGF/FGF-2 treatment does prime and stimulate EC tube responses to hematopoietic factors/FGF-2 which are supplied in the fibrin matrices. In contrast, reversing the order of addition where hematopoietic factors are added first as a priming stimulus followed by supplying VEGF/FGF-2 into the fibrin matrix during morphogenesis failed to provide a proper stimulus for EC tube formation and EC-pericyte tube co-assembly (Figure 5). Thus, VEGF primes EC tubulogenic responses to hematopoietic cytokines which are the critical growth factors that directly stimulate vascular morphogenesis in either collagen or fibrin matrices and under defined serum-free conditions (Figures 1,3,5) [16].

**Figure 6:**
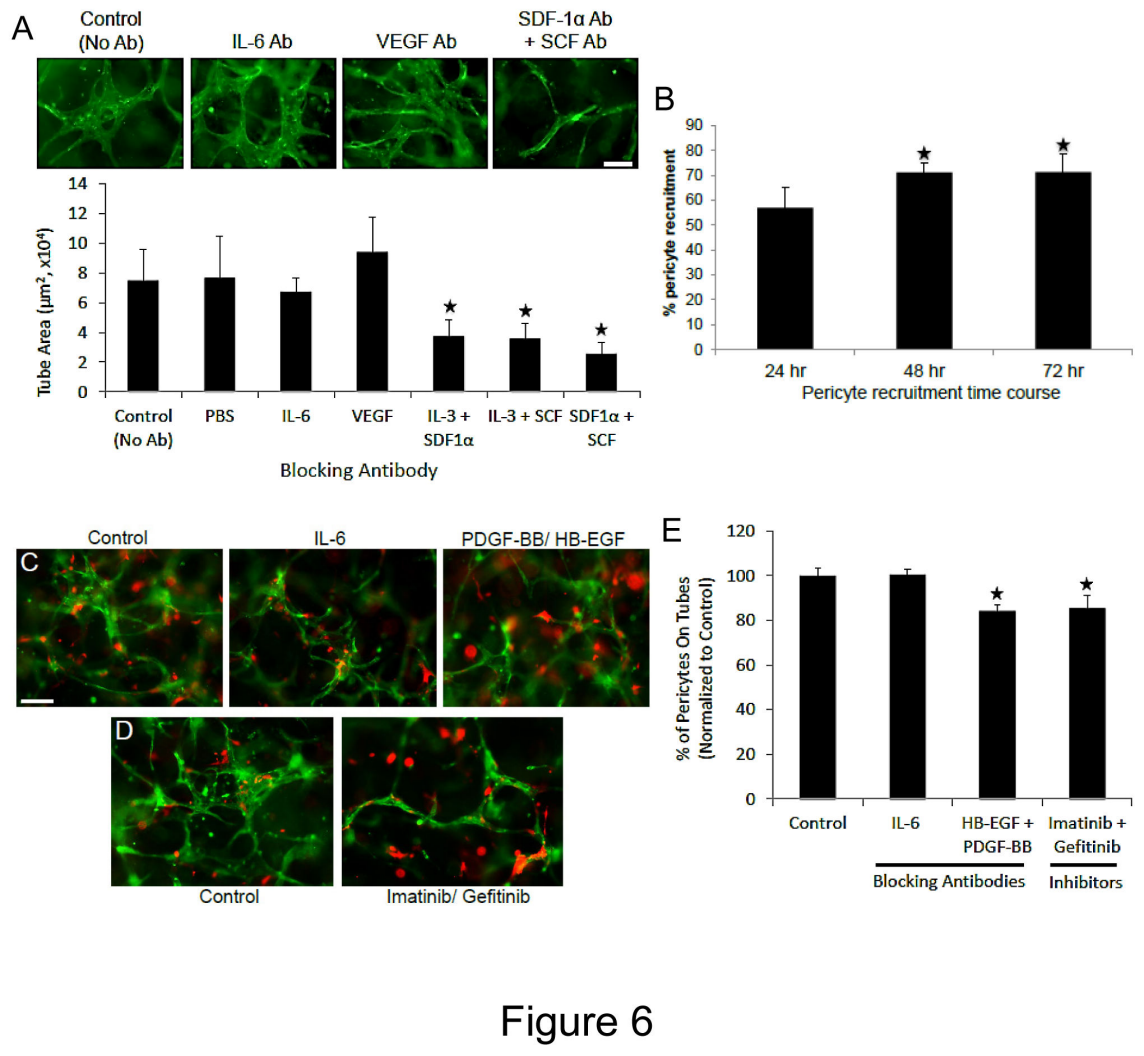
Neutralizing antibodies directed to the hematopoietic cytokines, SCF, IL-3 and SDF-1α markedly block EC tube formation, while antibodies to PDGF-BB and HB-EGF interfere with pericyte assembly on EC-lined tubes in 3D fibrin matrices under serum-free defined conditions. GFP-ECs were primed with hematopoietic cytokines (factors) or VEGF/FGF-2 overnight and then were seeded into fibrin matrices with hematopoietic factors and FGF-2 in the presence of Cherry-pericytes and in the presence of the indicated neutralizing antibodies or chemical inhibitors. (A) The antibodies were added at 50 µg/ml (IL-6, SCF, IL-3, SDF-1α) or 100 µg/ml (VEGF) versus controls and after 72 hr, cultures were fixed, photographed and quantitated for EC tube formation (n=10, values are averaged + SD, asterisks indicate significance compared to control, p<.01). (B) Time course of pericyte recruitment to EC tubes using real-time movies. At the indicated times, the percentage of pericytes that were associated with EC tubes were quantitated (n=8, values are averaged + SD, asterisks indicate significance compared to control, p<.01). (C,D) EC-pericyte co-cultures were established in the absence or presence of the indicated blocking antibodies (each added at 50 µg/ml) or chemical inhibitors (each added at 1 µM). After 72 hr, cultures were fixed and photographed (C,D) or were quantitated for pericyte recruitment (E) (n=6, values are averaged + SD, asterisks indicate significance compared to control, p<.01). Bar equals 100 µm.

To further characterize the growth factor dependence of the EC-pericyte tube co-assembly process, we performed blocking antibody and time course experiments. We show that the addition of neutralizing antibodies directed to SCF, IL-3 and SDF-1α markedly block EC tube formation in this system, while in contrast neutralizing antibodies directed to VEGF or IL-6 had no affect relative to control conditions (Figure 6A). This data strongly demonstrates the dependence of this EC-pericyte tube co-assembly process on hematopoietic stem cell cytokines in 3D fibrin matrices under defined conditions. A key further point is that the system also depends on the addition of recombinant hematopoietic cytokines as we demonstrate in Figures 1-5. In addition, we determined that pericyte recruitment begins as early as 24 hr of co-culture and appears to reach a maximum by 48 hr where about 70% of pericytes can be found associated with EC-lined tubes which then persists over time (Figure 6B) (Videos S1-S4). It should be re-emphasized that ECs and pericytes are randomly mixed in 3D fibrin matrices at 0 hr and there is no initial connection between ECs and pericytes, while by 24 hr, more than 50% of pericytes are observed to be associated with tubes (Figure 6B). This can also be observed in real-time Videos 1-3. Also, pericytes are recruited in a polarized fashion along the EC tube abluminal surface and are positioned to influence extracellular matrix remodeling events as discussed below. To address a role for growth factors on pericyte recruitment to EC tubes in 3D fibrin matrices, we added neutralizing antibodies to both PDGF-BB and HB-EGF as well as added chemical inhibitors of PDGFRbeta (Imatinib) and EGFR (Gefitinib) (Figure 6C-E). The data indicate that interfering with these ligands or receptors in combination results in significant blockade of pericyte association with EC tubes (Figure 6C-E). Thus, PDGF-BB and HB-EGF play a role in EC-pericyte tube co-assembly in either 3D fibrin or collagen matrices under serum-free defined conditions in a process that is stimulated by hematopoietic stem cell cytokines and FGF-2. 

### Hematopoietic stem cell cytokines and FGF-2 stimulate EC-pericyte tube co-assembly and vascular basement membrane matrix formation in 3D fibrin matrices under serum-free defined conditions

**Figure 7:**
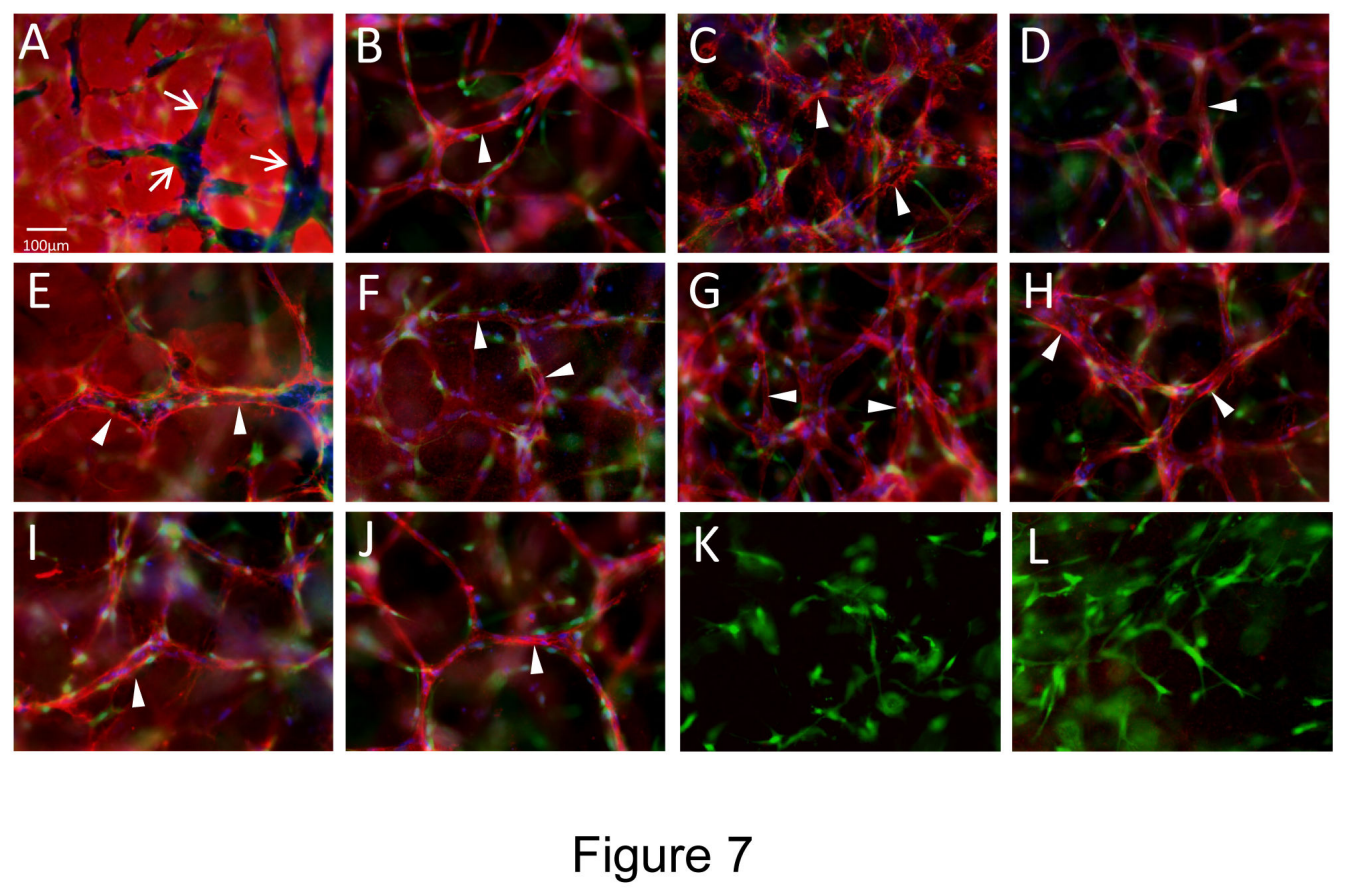
Hematopoietic stem cell cytokines stimulate EC-pericyte tube co-assembly and vascular basement membrane matrix deposition under serum-free defined conditions in 3D fibrin matrices. ECs were primed with VEGF/FGF-2 overnight and then were seeded with GFP-pericytes into 3D fibrin matrices containing SCF, IL-3, SDF-1α, Flt-3L, and FGF-2. After 120 hr, cultures were fixed and stained for extracellular antigens using various antibodies directed to: (A) Fibrin; (B) CD31; (C) Collagen type IV; (D) Laminin; (E) Fibronectin; (F) Perlecan; (G) Nidogen-1; (H) Nidogen-2; (K) Collagen type I; and (L) Collagen type III. In some cultures, the fibrin gel was supplemented with bovine fibronectin (I, J) and a monoclonal antibody that is specific for human fibronectin was used to assess fibronectin deposition within the vascular basement membrane (I). In this case, CD31 antibody staining was shown for comparison (J). In all other cases, the fibrin gels were supplemented with human fibronectin (A-H, K,L). Arrows indicate the borders of vascular guidance tunnels which are generated during EC tubulogenesis and both ECs and pericytes reside within these tunnel spaces (A). Arrowheads indicate vascular basement membrane deposition. Bar equals 25 µm.

**Figure 8:**
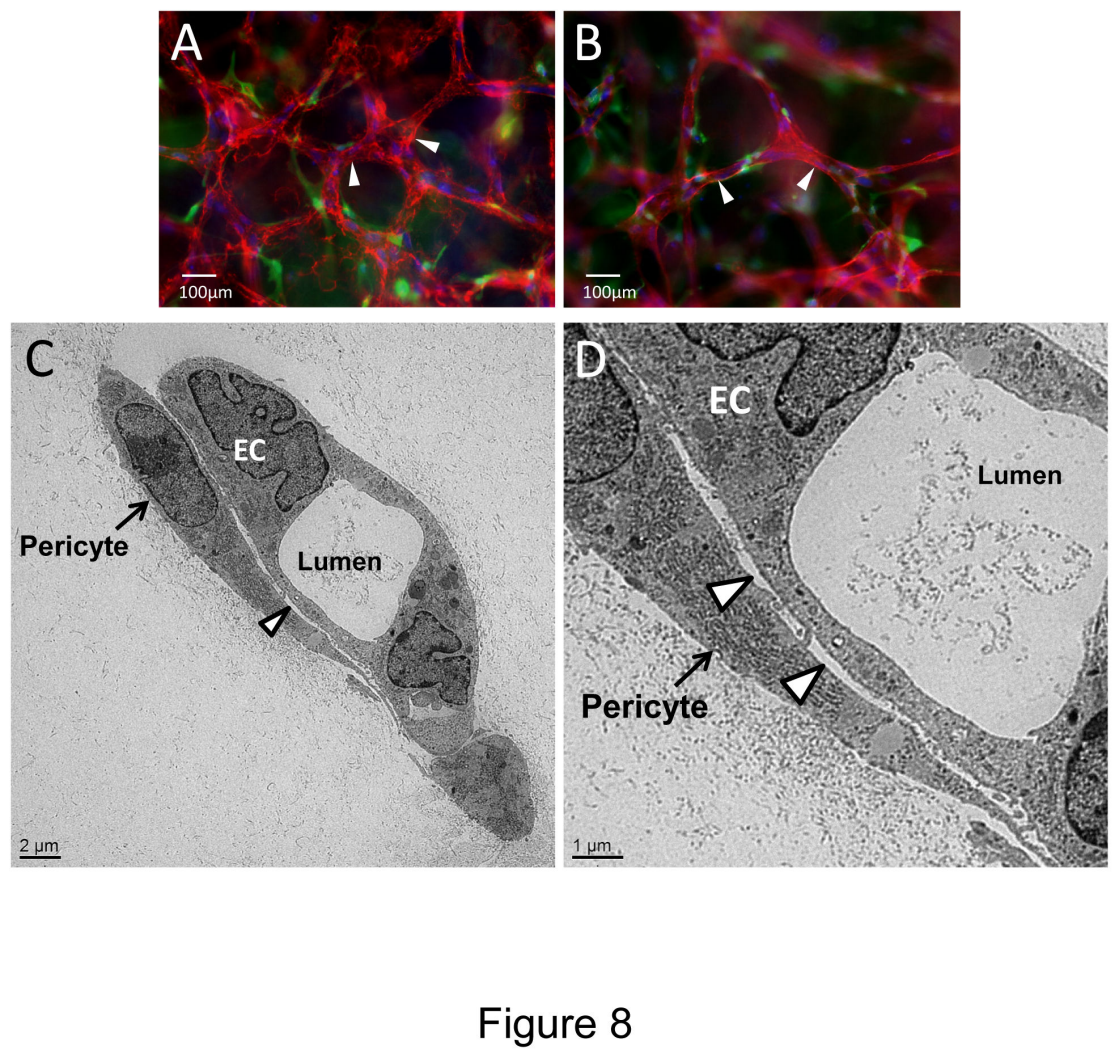
EC-pericyte tube co-assembly in 3D fibrin matrices under defined serum-free conditions leads to vascular basement membrane matrix formation. ECs that were primed with VEGF/FGF-2 and GFP-pericytes were seeded together into 3D fibrin matrices in the presence of hematopoietic stem cell cytokines and after 120 hr, cultures were fixed for fluorescent immunomicroscopy (A,B), and for transmission electron microscopy (C,D). Cultures were stained with antibodies to collagen type IV (A) and laminin (B) demonstrating vascular basement membrane formation. Arrowheads indicate basement membrane matrix deposition. Bar equals 100 µm. (C,D) Transmission electron micrographs showing pericytes along an EC tube structure with a defined lumen space and vascular basement membrane deposition observed between the two cell types (arrowheads). Arrows indicate pericytes. Bars equal 2 µm (C) or 1 µm (D).

 Previously, we reported that a major function of pericytes is to recruit to capillary tube networks and work together with ECs to control vascular basement membrane matrix assembly in 3D matrices [9,13,14]. This process occurs within vascular guidance tunnels, which are matrix spaces created by EC proteinase-dependent clearing of matrix during the tubulogenic process [45], and pericytes then recruit to EC tubes that reside within these spaces [9,13]. To address whether we can observe a similar outcome of EC-pericyte interactions in 3D fibrin matrices, we stained fixed co-cultures in the absence of detergent to assess the extracellular deposition of basement membrane components, fibrin, and interstitial collagens during these events (Figure 7). We demonstrate that EC tubulogenesis leads to the formation of vascular guidance tunnels (arrows) in fibrin matrices just like we have previously reported for collagen matrices [13,45] (Figure 7A). ECs generated these matrix spaces as a result of the tubulogenic process and after pericyte recruitment to EC tubes, both cell types are found within these tunnel spaces (Figure 7A). Staining for the vascular basement membrane constituents, laminin, fibronectin, collagen type IV, nidogen-1, nidogen-2, and perlecan were observed to deposit on the abluminal EC tube surface (arrowheads) as a result of EC-pericyte interactions within 3D fibrin matrices (Figures 7 and 8). For comparison, ECs are stained with CD31 and of interest is that there is no evidence for interstitial collagen deposition, as there was no apparent staining for type I or type III collagens despite evidence for strong expression of these collagens by pericytes at the mRNA level (data not shown). Finally, we performed transmission electron microscopy to further demonstrate that vascular basement membranes could be observed to deposit on the abluminal surface of EC tubes and in between ECs and pericytes (arrowheads) (Figure 8C,D). Thus, we have now demonstrated a number of key points regarding growth factor and matrix requirements as well as critical functional roles for ECs and pericytes during EC-pericyte tube co-assembly in 3D extracellular matrices. First, it is clear that hematopoietic stem cell cytokines and FGF-2 are required together in order for human EC-pericyte tube co-assembly to occur in either 3D collagen [16] or fibrin matrices under serum-free defined conditions (Figures 1-5). Second, EC-pericyte tube co-assembly leads to formation of the vascular basement membrane as assessed by either immunofluorescence staining (staining for extracellular deposition in the absence of detergents) or transmission electron microscopy in either 3D collagen [13] or fibrin matrices. Third, ECs and pericytes can be functionally defined by their respective, tubulogenic ability or ability to recruit to tubes in 3D matrices, and they clearly work together to affect EC tube remodeling events and vascular basement membrane matrix assembly in either type of 3D matrix environment. This new 3D fibrin model along with our previously described 3D collagen model, allow for a molecular dissection for how each of these events occur. 

### Hematopoietic stem cell cytokines and FGF-2 as well as pericytes markedly stimulate EC sprouting and tube morphogenesis in 3D fibrin matrices under serum-free defined conditions

**Figure 9:**
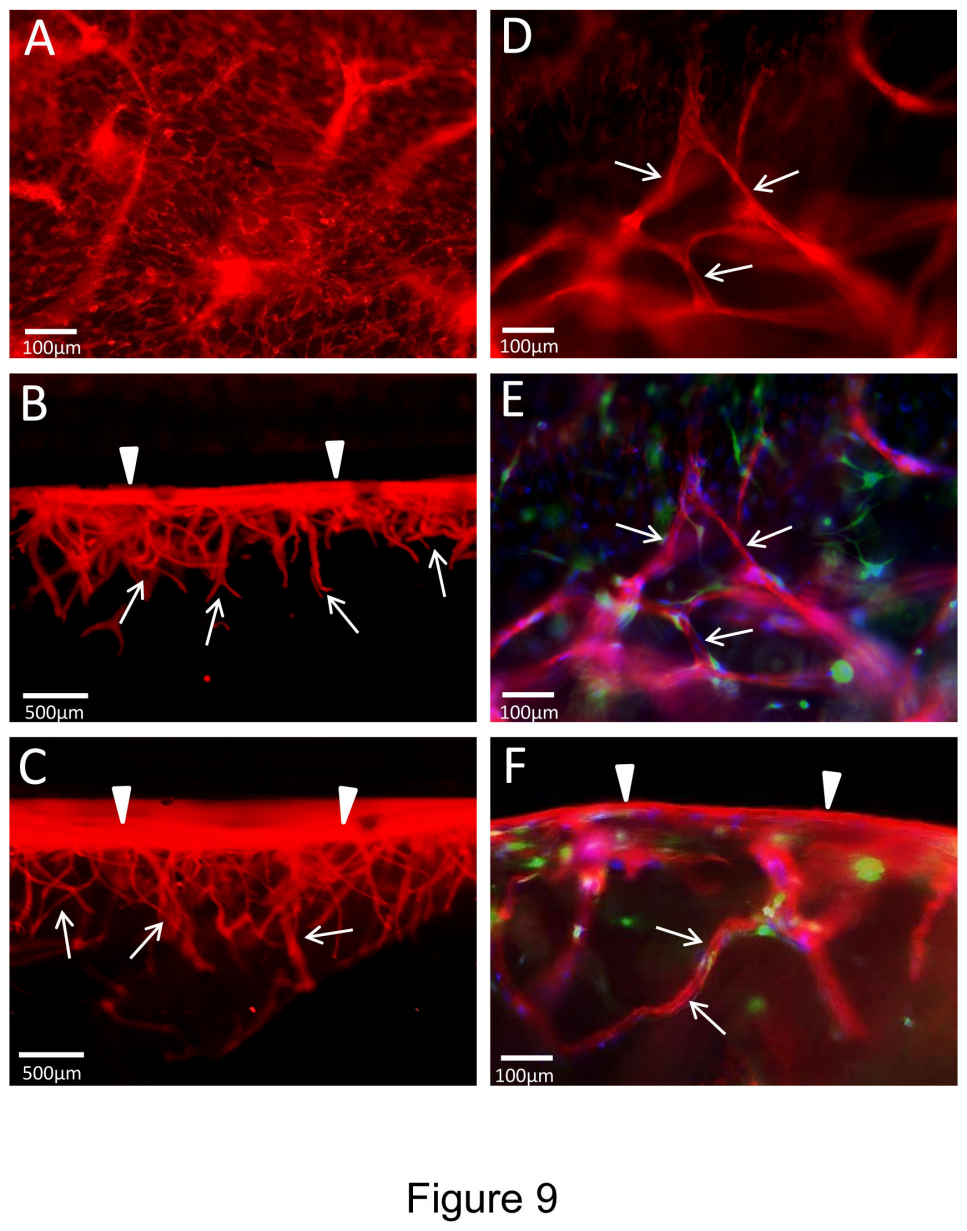
Hematopoietic stem cell cytokines, FGF-2 and pericytes stimulate EC sprouting and tube morphogenesis in 3D fibrin matrices under defined serum-free conditions. GFP-pericytes were seeded into 3D fibrin matrices which contained SCF, IL-3, SDF-1α, Flt-3L and FGF-2. ECs were primed with VEGF/FGF-2 and were seeded on the surface of the polymerized fibrin gels that contained growth factors and pericytes. ECs were allowed to sprout and form tubes for 120 hr prior to fixation and immunofluorescence staining with anti-CD31 antibodies (red). (A) Monolayer surface with visible EC-EC junctions as well as out of focus sprouts invading beneath the EC monolayer. (B,C) Cross sections of the cultures showing the monolayer surface (arrowheads) and invading EC sprouts and tubes (arrows). (D) Invading EC sprouts are observed (arrows) beneath the monolayer surface. (E) Overlay image of (D) showing EC sprouts (red) with associated and surrounding GFP-pericytes (green) and Hoechst dye-stained nuclei (blue). (F) Overlay image showing a cross-section of the EC monolayer (arrowheads) and invading EC sprouts (red) (arrows) with associated and surrounding GFP-pericytes (green) as well as Hoechst dye-stained nuclei (blue). Bar equals 100 µm (A,D,E,F) or 500 µm (B,C).

**Figure 10:**
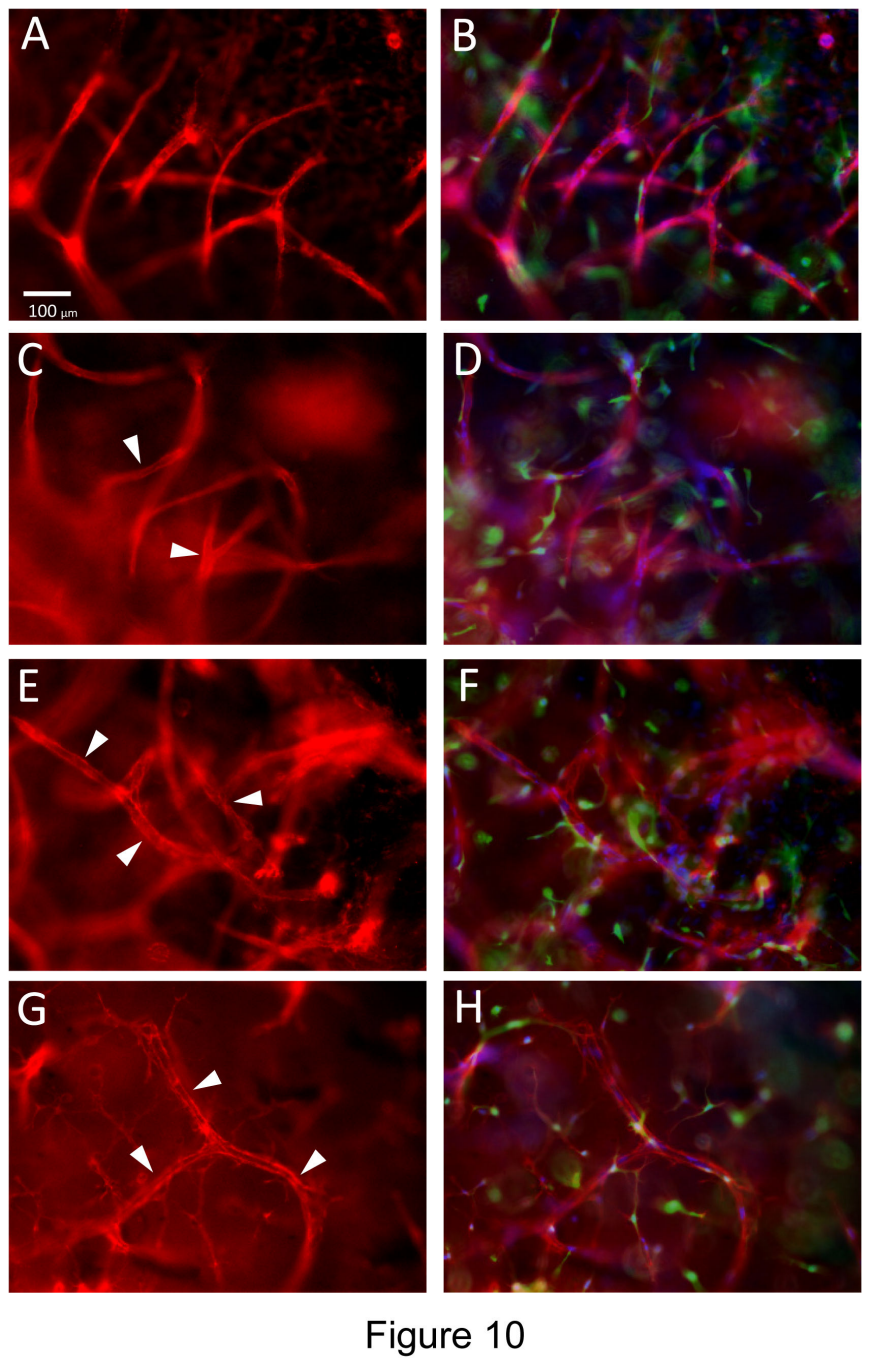
Pericytes recruit to invading EC sprouts/tubes and induce vascular basement membrane assembly on these EC tubes in 3D fibrin matrices under serum-free defined conditions. GFP-pericytes were seeded into 3D fibrin matrices which contained SCF, IL-3, SDF-1α, Flt-3L and FGF-2 while ECs were primed with VEGF/FGF-2 and then seeded on the surface of polymerized fibrin gels that contained growth factors and pericytes. ECs were allowed to sprout and form tubes for 120 hr prior to fixation and immunofluorescence staining with antibodies to CD31 (A,B); laminin (C,D); collagen type IV (E,F); or fibronectin (G,H). The basement membrane matrix antigens were stained in the absence of detergent to examine only extracellular deposition of these molecules. The right sided images (B,D,F,H) are overlay images for each stain showing the antigen stain in red, while GFP-pericytes are green and nuclei are blue (stained with Hoechst dye). Arrowheads indicate vascular basement membrane matrix deposition. Bar equals 100 µm.

**Figure 11:**
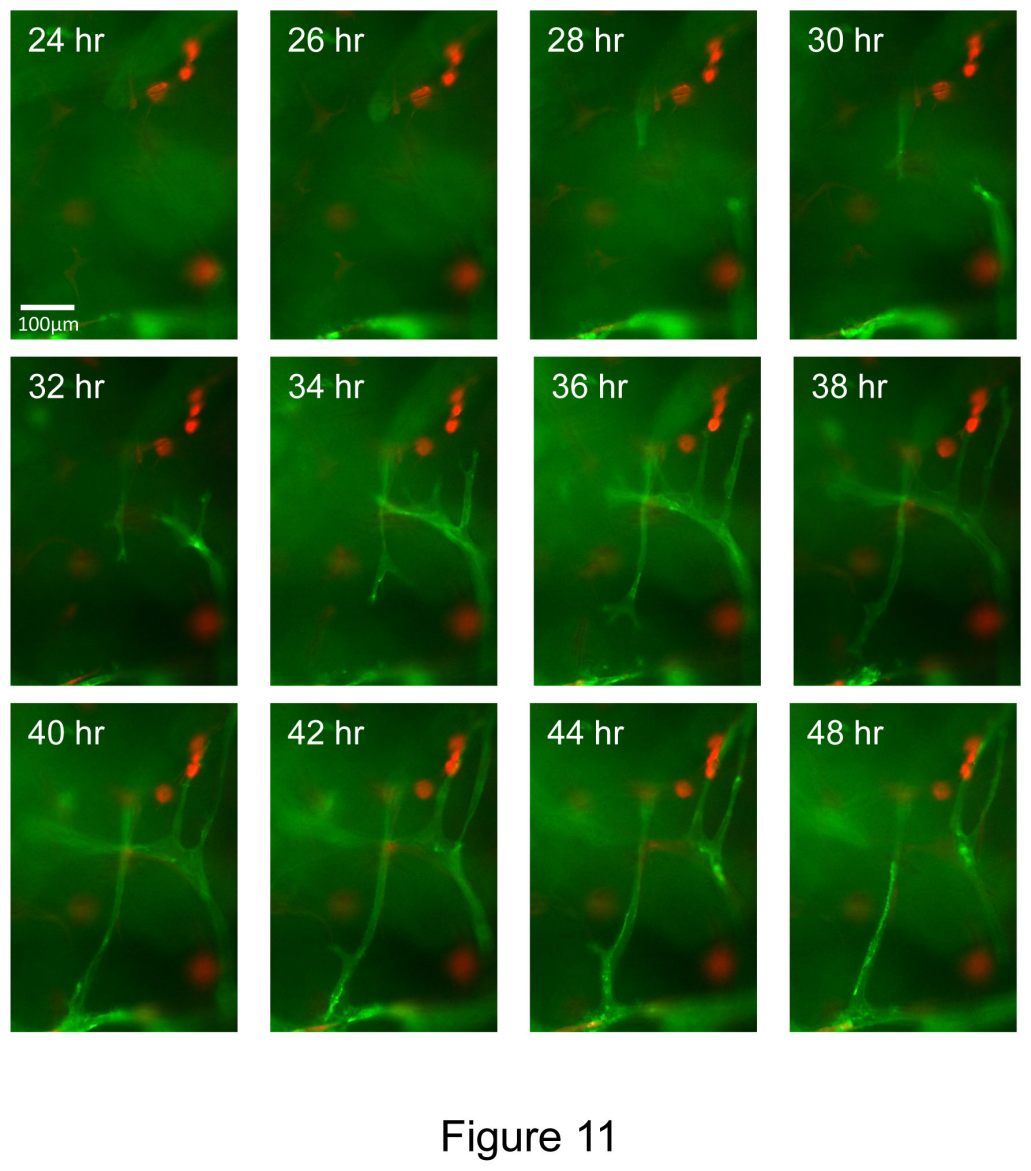
Time lapse images of EC sprouting and tubulogenesis in 3D fibrin matrices under serum-free defined conditions which are stimulated by hematopoietic stem cell cytokines, FGF-2 and pericytes. mCherry-pericytes, hematopoietic cytokines and FGF-2 were incorporated into the fibrin matrix, and VEGF/FGF-2 primed ECs (membrane AcGFP-labeled) were seeded onto the surface of fibrin gels and real-time movies were made. Select time points are shown from a representative field showing marked EC sprouting and tube formation as well as EC-pericyte interactions. Interestingly, it appears that EC sprouts might be preferentially orienting toward mCherry-pericytes in these movies suggesting the presence of pericyte-derived directional cues. Bar equals 100 µm.

A key morphogenic event during angiogenesis is sprouting from a monolayer surface and we have demonstrated this type of response using both collagen and fibrin matrices in previous work and *in vitro* models that we have described [16,18]. Here, we have examined if EC sprouting, tube formation and EC-pericyte recruitment as well as basement membrane assembly occur using our novel defined 3D fibrin model (Figures 9-11). In this assay, we incorporated the hematopoietic cytokines, FGF-2 and pericytes into the fibrin matrix and then seeded ECs onto the surface of these matrices. Over time, we were able to observe extensive EC sprouting with clear evidence for EC tip cells (Videos S5 and S6) and EC tube formation occurring as they invade over time (Figure 9). An EC monolayer surface (arrowheads) is clearly seen with CD31 positive junctional contacts (Figure 9A) and extensive EC sprouts (0.5- 1.0 mm in length) observed in cross-sections (arrows) (Figure 9B,C,F). Pericyte recruitment was also evident along these EC tube sprouts (Figures 9E,F and 10). Real-time movies of these events show marked EC sprouting with apparent tip cells (some of which are not connected to the trailing EC-lined tube) (Figure 11) (Videos S5 and S6). Of great interest is that EC sprouting (labeled with membrane-targeted AcGFP) appears to occur preferentially toward individual or small clusters of pericytes (labeled with mCherry) (Videos S5 and S6). This is highly suggestive that a directional cue is provided by pericytes during this EC invasion event. Finally, pericyte recruitment occurs in conjunction with vascular basement membrane assembly along the developing tubes in this sprouting system (Figure 10). 

### Integrin α5β1 plays a key role in EC-pericyte tube co-assembly and EC sprouting in 3D fibrin matrices under serum-free defined conditions

**Figure 12:**
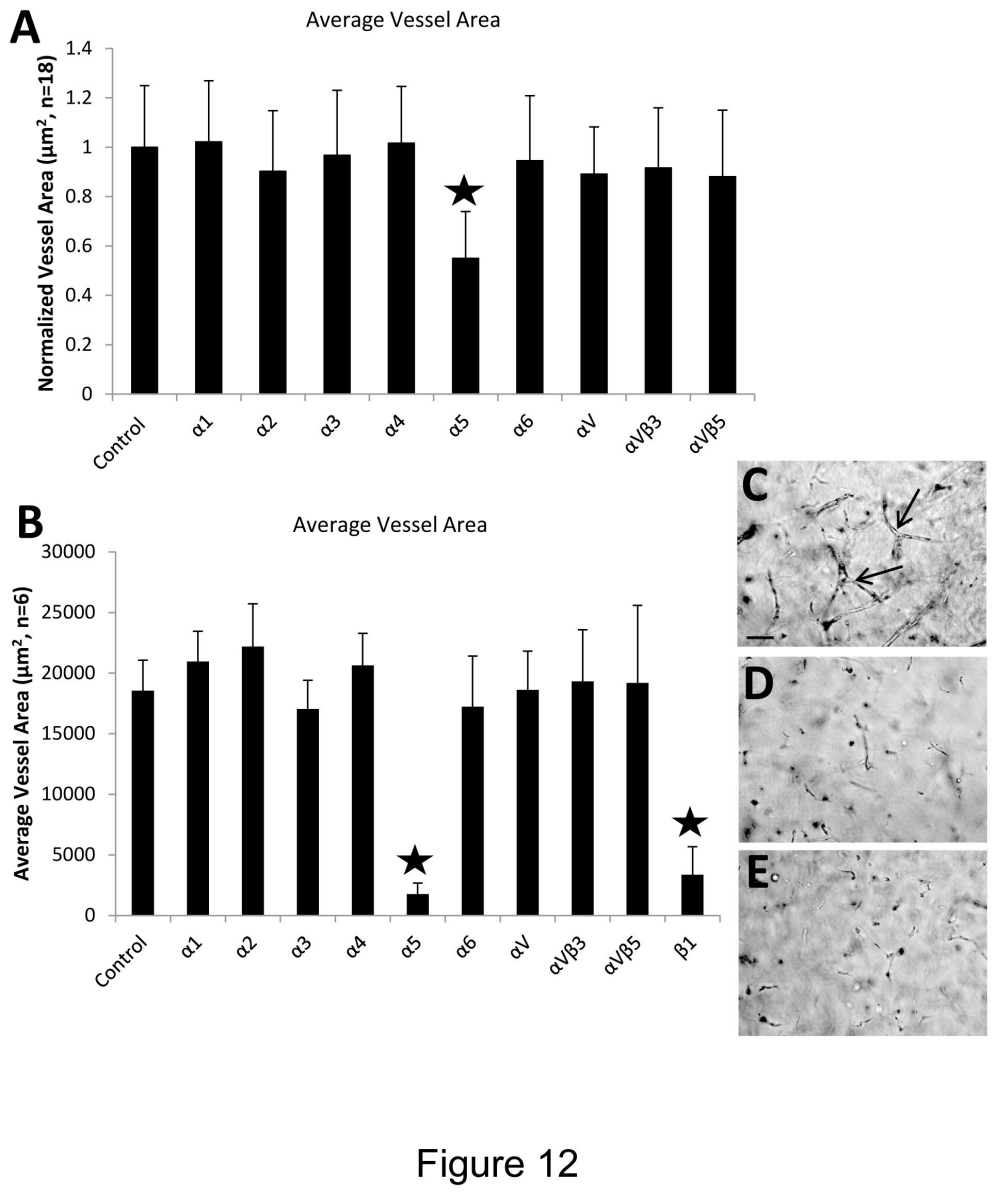
Integrin α5β1 controls EC sprouting and tubulogenesis in 3D fibrin matrices under serum-free defined conditions. (A) EC-pericyte co-cultures were established by suspending both cell types into fibrin matrices along with hematopoietic cytokines and FGF-2. The indicated anti-integrin blocking antibodies were added at 20 µg/ml and after 72 hr, EC tube area was determined using Metamorph software. Asterisk indicates significance at p< .01 compared to the control condition (n=18, values are averaged + SD). (B) EC sprouting assays were established by seeding ECs on the surface of fibrin gels which contained pericytes as well as hematopoietic cytokines and FGF-2. The indicated anti-integrin blocking antibodies were added at 20 µg/ml and after 72 hr, EC tube area in the invading sprouts was quantitated using Metamorph software. Asterisks indicate significance at p< .01 compared to the control condition (n=6, values are averaged + SD). Representative photographs of invading sprouts and EC tubulogenesis in the control condition (C); anti-α5 integrin subunit (D); and anti-β1 integrin subunit (E). Bar equals 100 µm.

 To identify which integrins appear to play the predominant role in the EC tube morphogenesis and sprouting processes, we utilized blocking antibodies directed to many of the known β1, β3 and β5 integrins. In both systems that were examined, the key integrin involved is the α5β1 integrin, a known receptor for fibronectin and fibrin matrices (Figure 12). In this serum-free defined system, there was no evidence for the involvement of αv integrins in these morphogenic responses. 

## Discussion

 In this work, we present a novel serum-free defined system in 3D fibrin matrices, whereby human ECs and pericytes co-assemble into capillary tube networks with lumen spaces and pericyte recruitment occurs along the tube abluminal surface. Furthermore, we demonstrated that EC-pericyte interactions in this model led to a critical blood vessel maturation event which is vascular basement membrane matrix deposition. A critical point is that we established this serum-free system so that we could define which growth factors were required for human blood vessel formation in a 3D matrix environment such as fibrin. We focused on 3D fibrin matrices which are an important provisional matrix that assembles during tissue injury responses so it is particularly relevant in postnatal life as well as in various disease states [8,44]. A fundamental conclusion of our studies is that the hematopoietic stem cell cytokines, SCF, IL-3 and SDF-1α are required for the ability of human ECs to form tubes in this 3D fibrin matrix environment. The addition of VEGF and FGF-2 together, which are generally considered to be the factors that control angiogenesis, are incapable of stimulating ECs to form tube networks under these serum-free defined conditions. However, our data does show that FGF-2 is important to be present along with hematopoietic cytokines to stimulate these processes. Thus, this work strongly suggests that the major factors which stimulate human vascular tube morphogenesis in a 3D fibrin matrix are SCF, IL-3, SDF-1α and FGF-2. Interestingly, we also report that an additional hematopoietic cytokine, Flt-3L, also adds to this response in this system. Another key point is that under these serum-free defined conditions, pericytes recruit to developing EC-lined tubes and together with ECs facilitate the assembly of the vascular basement membrane. Thus, this new model recapitulates what is known to occur within capillary tubes *in vivo* where EC-lined tubes have associated pericytes and a basement membrane lining the abluminal tube surface in 3D matrices [8,9,13]. 

 This new work coupled with our previously published findings further make the point that it is critical to define the specific growth factors that stimulate both human EC tubulogenesis and pericyte recruitment to EC-lined tubes. It is very important to understand both the growth factor requirements, but also the molecular signaling events that underlie the influence of these growth factors. Using our 3D collagen matrix model, we previously reported that the hematopoietic cytokines, SCF, IL-3 and SDF-1α along with FGF-2 were necessary for EC tubes to form under serum-free defined conditions [16]. The same factor combination works well in fibrin matrices under serum-free defined conditions as reported in the new work presented here. Thus, the combination of hematopoietic stem cell cytokines and FGF-2 stimulate vascular tube morphogenesis and maturation events in either 3D collagen or fibrin matrices [16]. Importantly, the combination of VEGF and FGF-2 together under the same serum-free conditions, fails to stimulate vascular tube formation or support pericyte recruitment to these developing tubes in either matrix environment [16]. Using the 3D collagen matrix system, we previously reported a critical role for EC-derived PDGF-BB and HB-EGF which together control pericyte recruitment, proliferation and basement membrane matrix assembly [14]. We observed that the presence of ECs was necessary in order for pericytes to migrate in 3D collagen matrices and also for them to proliferate during these processes and both events appeared to involve PDGF-BB and HB-EGF [14]. Here, we also show that PDGF-BB and HB-EGF play a role in EC-pericyte tube co-assembly in 3D fibrin matrices. In addition, we addressed the integrin requirements for EC tubulogenesis and sprouting in this defined fibrin model and show that the α5β1 integrin appears to be the major integrin responsible for these responses. Interestingly, we did not observe a role for αvβ3 integrin in these assays, perhaps because no serum was added which would have contributed vitronectin, a major adhesion protein present in serum. Our previous work showed a dual involvement of α5β1 and αvβ3 in EC lumen formation in 3D fibrin matrices, but this system utilized serum in the culture media [35].

Thus, the use of serum-free defined systems for both EC tubulogenesis and EC-pericyte tube co-assembly has led to our ability to define the specific role of particular growth factors which control EC or pericyte function during these processes. In addition, they have been critical to define the functional role of ECs vs. pericytes during capillary tube assembly in 3D matrices. ECs are defined by their markers, specific gene expression and anatomical position lining blood vessel walls, but a frequently overlooked issue is that a primary function of ECs is to form tube networks and, thus, the elucidation of mechanisms that control their tubulogenic ability (to both form and maintain tube structures) is of paramount importance [1,2,7]. Furthermore, there are considerable efforts ongoing to generate ECs using induced pluripotent stem cell (iPS) technology [46]. A critical requirement to demonstrate that iPS-generated ECs have the functional ability of isolated ECs, is that they must be able to generate tubes in a 3D assay system such as we describe here. A similar argument needs to be made regarding pericytes and their functions. Most studies describe pericytes by their anatomical position and their markers without addressing their functional abilities [9,47]. Our recent work clearly demonstrates that a key function of pericytes is to recruit to capillary tubes in 3D matrices through PDGF-BB and HB-EGF and to work with ECs through dynamic interactions (as illustrated by real-time movies shown here and in previous work), to construct the vascular basement membrane [9,13,14]. This work describes pericytes through their functional ability and the new serum-free defined fibrin system presented here allows for a functional test for a given cell type to determine whether it behaves like a pericyte. An intriguing issue that remains is why ECs generate tubes and pericytes do not, but instead recruit to the abluminal tube surface and then migrate along this surface. The novel 3D fibrin matrix system presented here represents a critical functional assay system to be able to answer such fundamental questions in molecular terms.

## Supporting Information

Video S1 **Real-time video of EC-pericyte tube co-assembly in 3D fibrin matrices under serum-free defined conditions.** ECs were labeled with membrane-targeted GFP and pericytes were labeled with mCherry. ECs were mixed with pericytes in fibrin matrices with added hematopoietic stem cell cytokines and FGF-2. ECs were primed with VEGF/FGF-2 overnight. The video shows EC tube assembly and pericyte recruitment to tubes from 0-72 hr. The video is shown at 16 frames/sec.
(MPG)

Video S2 **Real-time video of EC-pericyte tube co-assembly in 3D fibrin matrices under serum-free defined conditions.** ECs were labeled with membrane-targeted GFP and pericytes were labeled with mCherry. ECs were mixed with pericytes in fibrin matrices with added hematopoietic stem cell cytokines and FGF-2. ECs were primed with VEGF/FGF-2 overnight. The video shows EC tube assembly, pericyte recruitment to tubes, and pericyte motility along EC-lined tubes from 24-72 hr. The video is shown at 10 frames/sec.
(MPG)

Video S3 **Real-time video of EC-pericyte tube co-assembly in 3D fibrin matrices under serum-free defined conditions.** ECs were labeled with membrane-targeted GFP and pericytes were labeled with mCherry. ECs were mixed with pericytes in fibrin matrices with added hematopoietic stem cell cytokines and FGF-2. ECs were primed with VEGF/FGF-2 overnight. The video shows EC tube assembly, pericyte recruitment to tubes, and pericyte motility along EC-lined tubes from 24-72 hr. The video is shown at 11 frames/sec.
(MPG)

Video S4 **Real-time video of EC-pericyte tube co-assembly and remodeling in 3D fibrin matrices under serum-free defined conditions.** ECs were labeled with membrane-targeted GFP and pericytes were labeled with mCherry. ECs were mixed with pericytes in fibrin matrices with added hematopoietic stem cell cytokines and FGF-2. ECs were primed with VEGF/FGF-2 overnight. The video shows EC tube remodeling, and pericyte motility along EC-lined tubes from 72-120 hr. The video is shown at 12 frames/sec.
(MPG)

Video S5 **Real-time video of EC sprouting, tube formation and pericyte recruitment to tubes in 3D fibrin matrices under serum-free defined conditions.** ECs were labeled with membrane-targeted GFP and pericytes were labeled with mCherry. Pericytes were mixed in fibrin matrices with added hematopoietic stem cell cytokines and FGF-2. ECs were primed with VEGF/FGF-2 overnight and were seeded on the surface of the fibrin gels. The video shows EC sprouting which in many cases appears directed toward pericytes, EC tube formation, pericyte recruitment to tubes, and pericyte motility along EC-lined tubes from 24-48 hr. The video is shown at 12 frames/sec.
(MPG)

Video S6 **Real-time video of EC sprouting, tube formation and pericyte recruitment to tubes in 3D fibrin matrices under serum-free defined conditions.** ECs were labeled with membrane-targeted GFP and pericytes were labeled with mCherry. Pericytes were mixed in fibrin matrices with added hematopoietic stem cell cytokines and FGF-2. ECs were primed with VEGF/FGF-2 overnight and were seeded on the surface of the fibrin gels. The video shows EC sprouting which in many cases appears directed toward pericytes, EC tube formation, pericyte recruitment to tubes, and pericyte motility along EC-lined tubes from 24-48 hr. The video is shown at 8 frames/sec.
(MPG)
